# Occupational Dental Noise and Early Cochlear Changes: Evidence from Distortion Product Oto-Acoustic Emissions in Young Dentists

**DOI:** 10.3390/healthcare14070886

**Published:** 2026-03-30

**Authors:** Vijaya Kumar Narne, Ahmed A. Al-Bariqi, Ali Fahad Al-Qahtani, Krishna Yerraguntla, Praveen Prakash, Sreeraj Konadath, Reesha Oovattil Hussain, Shreyas Tikare, Mshari Nasser Alzidane, Budur Khalid Alsaanah

**Affiliations:** 1 Department of Medical Rehabilitation Sciences, College of Applied Medical Sciences, King Khalid University, Abha 61481, Saudi Arabiarehussain@kku.edu.sa (R.O.H.); 2 Speech-Language Pathology Unit, College of Applied Medical Sciences, King Khalid University, Abha 61481, Saudi Arabia; 3 Department of Periodontics and Community Dental Sciences, College of Dentistry, King Khalid University, Abha 61481, Saudi Arabia; aaalbarqi@kku.edu.sa (A.A.A.-B.);; 4 Department of Audiology, JSS Institute of Speech and Hearing, Dharwad 580007, India; praveenprakashp1849@gmail.com

**Keywords:** occupational noise exposure, otoacoustic emission, dental procedures

## Abstract

**Highlights:**

**What are the main findings?**

- A significant reduction in DPOAE amplitudes was observed immediately after clinical exposure in all participants, confirming an acute effect on outer hair cells.
- The immediate reduction in DPOAE amplitudes was significantly more pronounced in dentists who also used PLDs, compared to those exposed to occupational noise alone.

**What are the implications of the main findings?**

- The transient DPOAE changes validate this method as a sensitive tool for early detection of cochlear stress in dental professionals, enabling proactive hearing conservation before permanent loss develops.
- The heightened cochlear response in PLD users demonstrates that ototoxic risk is cumulative, meaning recreational listening habits must be considered alongside occupational exposure when evaluating auditory vulnerability.

**Abstract:**

**Background:** Dental professionals are routinely exposed to occupational noise from high-speed handpieces and ultrasonic scalers, with levels that can reach up to 90 dB(A). While such exposure is suspected to affect cochlear function, objective assessments in this population remain limited. This study investigated short-term changes in distortion product otoacoustic emissions (DPOAEs) as a biomarker of outer hair cell (OHC) function following routine clinical dental procedures. **Methods:** DPOAEs were recorded at frequencies from 1000 to 6000 Hz in young dental professionals with clinically normal hearing. Measurements were obtained at three time points: prior to dental procedures (baseline), immediately after exposure (3–5 min post-procedure), and at a 48-h (follow-up). Participants were stratified into two groups based on exposure profile: those exposed to occupational dental noise alone (Group 1) and those with concurrent use of personal listening devices (PLDs) in addition to occupational exposure (Group 2). **Results:** A significant reduction in DPOAE amplitudes was observed immediately following dental procedures in both groups, indicating an acute effect on OHC function. This reduction was more pronounced in Group 1 (PLD users) compared to Group 2 (occupational noise only). Amplitudes returned to baseline levels at the 48-h follow-up in both groups, confirming the transient nature of the effect. The absence of significant Frequency × Time interactions indicates that the observed amplitude reductions were broadly distributed across the tested frequency range rather than confined to a specific spectral region. **Conclusions:** Routine clinical dental procedures can induce transient, measurable changes in cochlear outer hair cell function, detectable by DPOAEs in young professionals with normal audiometric thresholds. Although these changes appear reversible within 48 h, the greater acute response observed in individuals with concurrent personal listening device use suggests that cumulative acoustic exposure may increase cochlear susceptibility. These findings support the integration of objective cochlear monitoring into occupational health surveillance for dental personnel.

## 1. Introduction

Occupational noise exposure remains a significant global public health concern and is recognised as one of the most prevalent workplace hazards worldwide. The World Health Organisation (WHO) estimates that over 1.5 billion people globally live with some degree of hearing loss, with a substantial proportion attributable to preventable environmental and occupational exposures [1]. Noise induced hearing loss (NIHL) continues to rank among the most common occupational diseases internationally, accounting for a considerable share of work related morbidity and long-term disability [2]. Although industrial and manufacturing sectors have traditionally received regulatory attention, hazardous noise exposure within healthcare settings remains comparatively under-recognised despite increasing evidence of risk.

Healthcare environments such as dental clinics generate intermittent noise levels capable of inducing temporary and permanent auditory effects. Dental professionals are routinely exposed to airborne acoustic energy and structure-borne vibration produced by high-speed hand-pieces, ultrasonic scalers, micro-motors, and suction systems [3,4]. Measured “A” weighted sound pressure levels (LAeq) at the operator’s ear frequently range between 80 and 97 dB SPL [3,4]. The simultaneous operation of multiple devices, such as a hand-piece and high-volume suction, can elevate the cumulative noise level to between 94 and 96 dB(A) [5,6]. These levels are of significant occupational concern when considered against international safety standards [7,8]. Applying the 3 dB exchange rate principle, permissible exposure duration is halved for every 3 dB increase in sound level. Consequently, while exposure at 88 dB(A) is limited to 4 h, the 94–96 dB(A) range associated with combined instrument use would limit safe, unprotected exposure to approximately 1 h, underscoring the potential occupational relevance of dental equipment noise. Available evidence suggests that active exposure to dental instruments ranges from 30 to 120 min per day. Under a 3-dB exchange rate (NIOSH), levels of 94–96 dB(A) correspond to permissible durations of 1 h to 30 min. However, dental noise exposure is intermittent, occurring in short, task-dependent bursts rather than continuously. Accordingly, cumulative exposure is better represented by time-weighted average (TWA) metrics. Personal dosimetry studies report 8 h TWA levels of 60–75 dB(A), including values of 64.3–68.9 dB(A) in dental settings, despite transient peaks exceeding 120–130 dB. Thus, even when average exposure remains below occupational limits, repeated high-intensity peaks and cumulative dose may still pose a risk to cochlear function [9,10].

Although dental noise exposure is often intermittent, cumulative daily exposure over years of clinical practice may pose substantial auditory risk. A systematic review of dental professionals reported a positive association between years of clinical experience and hearing impairment, particularly at high frequencies [11]. Clinical surveys in paediatric dental settings have further shown that average operator noise levels exceed the 85 dB(A) recommended limit for a substantial proportion of practitioners [6]. Professional organisations, including the American Dental Association, have cautioned that even moderate but repeated exposure across a multi-decade career may contribute to noise-induced hearing loss and tinnitus [12].

Repeated daily exposure at these levels, even when below regulatory limits for industrial settings, may contribute to subclinical auditory dysfunction over time. Cochlear outer hair cells (OHCs) are essential for maintaining auditory sensitivity and frequency resolution [13,14]. Functional alterations in OHCs may occur before measurable threshold shifts are detected using conventional pure-tone audiometry. The WHO has emphasised that early cochlear changes may remain undetected when screening programs rely solely on behavioural threshold measures [1]. Otoacoustic emissions (OAEs), generated by OHC-related cochlear processes, provide a sensitive and objective indicator of cochlear function [15]. Distortion product otoacoustic emissions (DPOAEs), in particular, have demonstrated utility in detecting early exposure-related cochlear changes in individuals with clinically normal audiometric thresholds [16,17,18]. Both occupational and experimental studies have shown that short-term noise exposure can produce measurable reductions in DPOAE amplitudes even when permanent hearing loss is not evident [19,20,21]. Accordingly, DPOAEs serve as an early functional biomarker of cochlear status and may reveal exposure-related changes prior to clinically apparent threshold elevation.

In parallel, WHO reports that more than one billion young people worldwide are at risk of hearing loss due to unsafe recreational listening practices, particularly through personal listening devices (PLDs) [1,22]. Many young healthcare professionals regularly use PLDs at levels ranging between 75–90 dB(A) [23], creating the potential for additive exposure when combined with occupational dental noise. This combined or cumulative exposure represents an emerging concern within occupational epidemiology, particularly in younger workforces.

The present study focused on young dental practitioners to examine early cochlear changes in a population with relatively limited cumulative occupational exposure. This approach minimizes the confounding effects of long-term noise exposure, aging, and pre-existing auditory deficits, thereby allowing clearer identification of short-term, exposure-related changes in cochlear function. Young dental practitioners with clinically normal hearing thresholds typically exhibit robust outer hair cell (OHC) function, making them a sensitive model for detecting subtle, transient alterations using otoacoustic emissions [24,25]. In contrast, individuals with greater than 5 years of dental practitioners are more likely to exhibit permanent threshold shifts on audiometry, particularly at high frequencies, reflecting cumulative cochlear damage [9,25]. Moreover, experimental and translational studies have demonstrated that repeated noise exposure can induce cochlear changes that are not immediately reflected in audiometric thresholds but can be detected using objective measures such as otoacoustic emissions [26]. By selecting a younger cohort with shorter exposure histories, the study aims to capture early, potentially reversible changes in cochlear function prior to the development of measurable audiometric hearing loss.

From a healthcare systems standpoint, early identification of reversible cochlear stress is critical. Subclinical auditory dysfunction may compromise communication in clinical environments, increase cognitive listening effort, and, if cumulative, contribute to long-term hearing impairment and associated healthcare costs. The WHO advocates strengthening preventive and monitoring frameworks for hearing health within occupational settings as part of integrated primary healthcare models [1]. Distortion product otoacoustic emissions (DPOAEs) offer frequency-specific assessment of OHC function, demonstrate robust test–retest reliability in adults, and are less influenced by transient physiological variability compared to transient evoked OAEs [27,28]. Their sensitivity to early cochlear changes makes them particularly suitable for occupational hearing surveillance among healthcare professionals exposed to moderate but repeated acoustic stress.

Accordingly, the present study aimed to investigate acute and short-term changes in distortion product otoacoustic emission amplitudes measured before, immediately after, and 48 h following dental procedures in young dental doctors with clinically normal hearing sensitivity. Additionally, the study examined whether combined exposure to dental noise and regular personal listening device use influences the magnitude of cochlear response changes. By integrating objective cochlear assessment within a healthcare occupational framework, this study seeks to contribute evidence toward strengthening hearing conservation strategies in dental practice.

## 2. Materials and Methods

The study followed a prospective observational study with repeated measures. A total of 40 (20 male and 20 female) individuals participated in the study. All participants were dental practitioners (operators) and not patients receiving treatment and they were recruited from dental college, King Khalid University, Abha, Saudi Arabia.

### 2.1. Participants

The study involved a total of 40 participants (80 ears) in the age range of 19–26 years, grouped into two groups. Group-1 includes dentists without using PLD’s, and group-2 includes dentists using PLD’s. To minimise observer bias, the investigator analysing the DPOAE recordings was blinded to the participants’ group identity during data processing and statistical analysis. The sample size determination was conducted using the G*Power3.1.24. In the present study, “normative DPOAE” refers to emission amplitudes reported in individuals with normal hearing sensitivity and no otological pathology, as described in established literature [24]. Sample size estimation was informed by prior studies examining short-term noise exposure, which report small reductions in DPOAE amplitude (typically in the range of 1–3 dB) following acoustic exposure [29]. Based on these findings, the study was powered to detect small-to-moderate changes in DPOAE amplitude. A sample size of N = 40 was estimated (comprising both groups), aiming for a statistical significance (α) of 0.05 and a power (1−β) of 0.80. Thus, a sample size of 40 (20 in each group) was considered appropriate for this study.

#### 2.1.1. Group I: Dentists with Using PLD’s

The dentists using PLDs group consisted of 18 individuals (10 males, 8 females) aged 19 to 27 years (mean age: 23.88 ± 5.2 years). All participants had pure-tone thresholds below 15 dB HL at octave frequencies and 100% speech identification scores at 40 dB SL (re: Speech Recognition Threshold). Tympanometry results were normal (Type A), and both DPOAEs and TEOAEs were present. Participation in the study was entirely voluntary. PLD use was assessed using a structured self-report questionnaire administered prior to testing. Participants reported the frequency and duration of use, type of device, and typical listening volume. All subjects in this group reported regular PLD use, with an average daily usage of approximately 6–8 h. All participants predominantly used earphones for recreational listening.

Estimated listening levels (in dBA) were derived based on reported volume settings and corresponding ear-canal output levels as described in World Health Organization (WHO) guidelines [22]. The estimated output levels ranged from 80 to 90 dBA, with a majority of participants clustered around approximately 90 dBA.

#### 2.1.2. Group II: Dentists Without Using PLDs

The normal hearing (NH) group consisted of 22 individuals (11 males, 11 females) aged 19 to 25 years (mean age: 21.88 ± 3.2 years). All participants had pure-tone thresholds below 15 dB HL at octave frequencies and 100% speech identification scores at 40 dB SL (re: Speech Recognition Threshold). Tympanometry results were normal (Type A), and both DPOAEs and TEOAEs were present. The presence of DPOAEs was determined based on a signal-to-noise ratio (SNR) of at least 6 dB, along with an absolute DPOAE amplitude greater than −10 dB SPL. TEOAEs were considered present when reproducibility exceeded 70% and the SNR was at least 6 dB across the specified frequency bands. Participation in the study was entirely voluntary, and this group reported rare use of personal listening devices (PLDs).

#### 2.1.3. Ethical Considerations

The study involved non-invasive procedures conducted on dentists working at university dental clinic. Prior to enrolment, participants were informed in detail about the primary objectives of the study, the procedures involved, the duration required for testing, and the number and timing of repeated measurements. Written informed consent was obtained from all participants. Ethical clearance for the study was obtained from the King Khalid University ethical review committee, with approval number IRB/KKUCOD/ETH/2023-24/044.

### 2.2. Procedure

A detailed case history was obtained from all participants before audiological evaluation, with specific probing into the presence or history of hearing loss and any related otological symptoms. Individuals who reported any active condition or history of risk factors with potential audiological impact were excluded from the study.

All enrolled participants underwent pure-tone audiometry, which included air-conduction and bone-conduction threshold measurements at standard clinical audiometric frequencies (250–8k Hz). All participants demonstrated clinically normal hearing sensitivity, with audiometric thresholds and pure-tone averages (computed for test frequencies 500, 1k, 2k, and 4k Hz) less than 15 dB HL. Speech audiometry revealed speech identification scores 100%, and uncomfortable loudness level (UCL) measures were ≥100 dB HL for all participants. Middle ear function was assessed using 226 Hz probe tone tympanometry, which revealed Type A tympanograms bilaterally, along with the presence of both ipsilateral and contralateral acoustic reflexes in both ears at test frequencies of 500, 1k, 2k, and 4k Hz.

#### 2.2.1. Measurements of Noise Levels

Noise measurements were conducted in the clinical operatory (dental chair area) under semi-isolated conditions to minimize interference from ambient clinic noise. These measurements were obtained during the same dental procedures in which DPOAE recordings were performed. The primary target of the measurements was the operator (dentist), the noise levels of the equipment were measured in two microphone position are (1) noise level was measured at equipment where the microphone was kept 1 cm from equipment and (2) operator ear level, which is at a distance of 15 cm from a main noise source to simulate the auditory position of the operator (dentists). The noise levels were measured over entire dental procedure. The same procedure was repeated 6 times sequentially in the same day, giving a total of recorded 6 measurements for each piece of equipment. The mean of the values was determined and the overall value was recorded. The noise levels for the ultrasonic scaler, turbine, contra angle hand-piece, micro motor hand-piece, low volume suction pump, high volume suction pump were measured in clinical areas. The measurements were taken with the equipment only turned on and without cutting.

The sound levels were measured with a precision sound level meter (BEHA UNITEST 93517, Germany). The noise measurement equipment (sound level meter, noise dosimeter, and acoustic calibrator) was calibrated using an external accredited laboratory to ISO/IEC 17,025 standards and issued calibration certificates. Sound levels were measured in A-weighted sound levels in decibels dB(A). The sound level is measured on the A scale, which was designed to mimic the response of the human ear.

#### 2.2.2. Measurement of DPOAEs

DPOAEs were recorded in a quiet cabin, proximal to the clinical area, using the Otoacoustics’ Titan system (firmware version 1.10.14). DP-grams were obtained at f2 frequencies of 1000, 1501, 2002, 3174, 4004, and 6384 Hz. Stimuli were presented at L1 = 65 dB SPL, L2 = 55 dB SPL, with an $\frac{f2}{f1}$ ratio of 1.22. The distortion product ($2f_{1}-f_{2}$) served as the outcome measure, providing frequency-specific information on outer hair cell function. For a DPOAE to be considered present and reproducible at a given f2 frequency, the typical criterion used for this type of research protocol is a signal-to-noise ratio (SNR) of >6 dB (meaning the DPOAE amplitude must be at least >6 dB above the noise floor). The $2f_{1}-f_{2}$ distortion product amplitude itself is commonly measured within a narrow frequency band (filter band), typically a $\frac{1}{2}$ or $\frac{1}{3}$ octave band centred around $2f_{1}-f_{2}$ frequency. The absolute DPOAE amplitude measurement was considered in dB SPL. DPOAE presence was defined using an SNR ≥ 6 dB. Absolute DPOAE amplitude (dB SPL) was used as the primary outcome measure for statistical analysis. This measure was preferred over the signal-to-noise ratio (SNR) because the study aimed to evaluate changes in cochlear output (outer hair cell function) independent of variations in background noise that can influence SNR.

**Figure 1:**
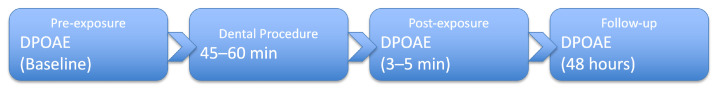
Experimental timeline of DPOAE measurements. Baseline (pre-exposure) DPOAEs were recorded prior to the dental procedure. The dental procedure lasted approximately 45–60 min, after which post-exposure DPOAEs were obtained within 3–5 min. A follow-up DPOAE assessment was conducted 48 h post-procedure.

Distortion product otoacoustic emissions (DPOAEs) were recorded at three time points. Baseline measurements (pre-exposure) were obtained prior to the dental procedure. Participants performed routine dental procedures as part of their clinical duties, with procedure durations varying between approximately 45 min and 1 h. Immediately following completion of the procedure, post-exposure DPOAE recordings were obtained within 3–5 min. Subsequently, all participants returned for a follow-up DPOAE assessment conducted 48 h after the dental procedure. This repeated-measures design allowed evaluation of immediate and short-term changes in cochlear outer hair cell function associated with dental procedure-related noise exposure. The experimental timeline of DPOAE measurements is given in Figure 1, below.

Distortion product otoacoustic emissions (DPOAEs) were recorded at three time points. Baseline measurements (Pre-exposure) were obtained prior to the dental procedure. Participants performed routine dental procedures as part of their clinical duties, with procedure durations varying between approximately 45 min and 1 h. Immediately following completion of the procedure, post-exposure DPOAE recordings were obtained within 3–5 min. Subsequently, all participants returned for a follow-up DPOAE assessment conducted 48 h after the dental procedure. The selection of these measurement intervals was based on the time-dependent nature of cochlear outer hair cell (OHC) responses to acoustic exposure. The immediate post-exposure measurement (3–5 min) was intended to capture acute, reversible changes associated with early metabolic stress, while the 48-h follow-up was chosen to evaluate recovery of OHC function and distinguish transient effects from potential longer-lasting alterations. Previous studies have demonstrated that noise-induced changes in otoacoustic emissions and cochlear function evolve over time and may recover within hours to days depending on exposure characteristics [26]. This repeated-measures design allowed evaluation of immediate and short-term changes in cochlear outer hair cell function associated with dental procedure-related noise exposure. The experimental timeline of DPOAE measurements is given in Figure 1 below.

### 2.3. Statistical Analysis

All statistical analyses were performed in R (version 4.4.0; R Core Team, Vienna, Austria, 2025). Mixed-effects modelling was performed using the lme4 package [30]. For DPOAE levels, a linear mixed-effects model (LMM) with a was fitted. The fixed-effects structure included group and time. To account for individual variability, a random intercept was specified for participant. The statistical significance of fixed effects was evaluated using Type III ANOVA with Satterthwaite approximation as implemented in the car package [31]. For the measured LEAeq levels of the noise at two locations, a paired-sample *t*-test was performed.

## 3. Results

### 3.1. Noise Levels of Dental Equipment

**Figure 2:**
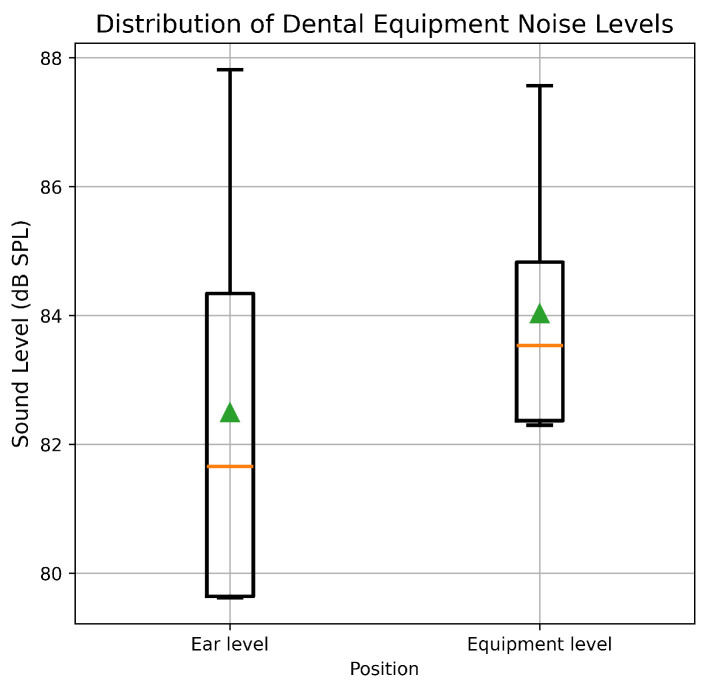
Box-plot represent the distribution of sound pressure levels (dB SPL) measured at the ear level and at the equipment level during dental procedures. The green triangle denotes the mean sound level for each position.

Figure 2 illustrates the mean noise levels (±1 SD) of dental equipment measured in the present study. The overall A-weighted equivalent continuous sound pressure level (LAeq) at the equipment level was 83.0 dB SPL, whereas the corresponding level at the operator’s ear position was 81.5 dB SPL. These values are comparable to those reported in earlier investigations of dental clinical noise exposure [11,32].

Comparison of noise levels measured at the equipment level and at ear level indicated modest attenuation with distance; equipment-level LAeq values varied within ±3 dB, whereas ear-level measurements varied within ±4 dB across repeated trials. Statistical comparison of six paired measurements revealed no significant difference between equipment-level and ear-level noise exposure (paired *t*-test, *p* > 0.05).

### 3.2. DPOAE Levels Before Exposure and After Exposure

**Figure 3:**
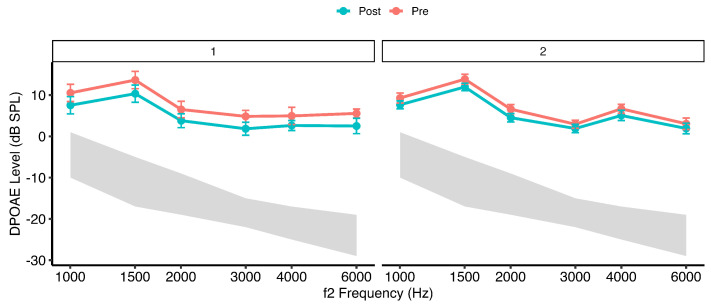
Mean distortion product otoacoustic emission (DPOAE) levels (DP level; dB SPL) as a function of f2 frequency in dental practitioners (dentists), measured before (Pre) and after (Post) performing routine clinical procedures. Results are shown for Group 1 (occupational dental noise exposure only) and Group 2 (combined occupational and personal listening device exposure). Solid lines represent group means, and shaded regions denote 95% confidence intervals.

**Figure 4:**
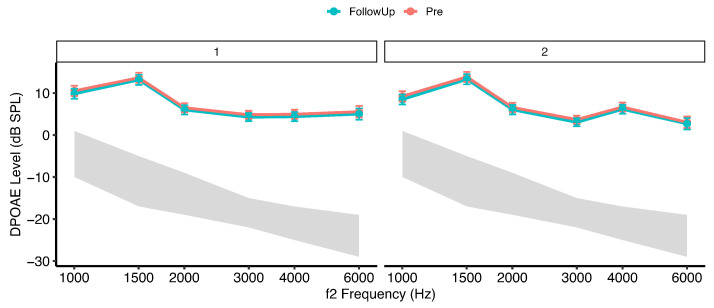
Same as Figure 3 with pre-exposure and follow-up.

Figure 3 and Figure 4 illustrate mean distortion product otoacoustic emission (DPOAE) levels (±95% confidence intervals) across $f_{2}$ frequencies measured at pre-exposure, post-exposure, and follow-up time points in the two groups. At baseline (pre-exposure), DPOAE amplitudes were comparable between Group 1 (dentists exposed to dental equipment noise only) and Group 2 (dentists additionally using personal listening devices), indicating similar outer hair cell (OHC) function prior to exposure.

Immediately following the dental procedure (post-exposure), both groups demonstrated a reduction in DPOAE amplitudes, consistent with acute exposure-related cochlear stress. The magnitude of reduction was greater in Group 2 than in Group 1. Across frequencies, the most prominent decreases were observed in the mid-to-high $f_{2}$ range (approximately 3–6 kHz). At the follow-up assessment (48 h), DPOAE levels in both groups returned to values comparable to baseline, demonstrating recovery of cochlear responses.

A linear mixed-effects model with subject-specific random intercepts (Type III ANOVA, Satterthwaite approximation) revealed a significant main effect of frequency, F(5, 527.15)=10.87, p<0.001, $\eta_{p}^{2}=0.09$, indicating moderate frequency-dependent variation in DPOAE amplitudes. A significant main effect of time was also observed, F(2, 526.00)=10.95, p<0.001, $\eta_{p}^{2}=0.04$, reflecting small-to-moderate exposure-related changes across measurement intervals. The main effect of group was not statistically significant, F(1, 528.54)=1.29, p=0.26, suggesting comparable overall DPOAE amplitudes between groups when averaged across time and frequency.

Importantly, a significant Group×Time interaction was identified, F(2, 526.00)=3.37, p=0.035, $\eta_{p}^{2}=0.01$. Although the associated effect size was small, this interaction indicates differential temporal response patterns between groups. No significant Group×Frequency, Frequency×Time, or three-way interactions were observed (all p>0.80), suggesting that temporal exposure effects were broadly consistent across the tested frequency range.

Post hoc comparisons of estimated marginal means (Kenward–Roger degrees of freedom, Tukey-adjusted) averaged across $f_{2}$ frequencies revealed significant Pre–Post reductions in DPOAE amplitudes in both groups. In Group 1, DPOAE levels decreased significantly from pre- to post-exposure (mean difference = 3.12 dB, SE = 0.55, t(526)=5.69, p<0.0001). In Group 2, a smaller but statistically significant reduction was observed (mean difference = 1.58 dB, SE = 0.40, t(526)=3.93, p=0.0003).

Comparisons between pre-exposure and follow-up measurements demonstrated recovery of cochlear function in both groups. In Group 1, follow-up levels did not differ significantly from baseline (mean difference = −0.17 dB, p=0.949), and in Group 2, no significant pre-to-follow-up difference was observed (mean difference = −0.21 dB, p=0.862). Significant post-to-follow-up contrasts in both groups confirmed reversibility of the acute reduction (both p<0.0001).

The intraclass correlation coefficient (ICC) indicated substantial between-subject variability in DPOAE amplitudes. The adjusted ICC was 0.695, suggesting that approximately 70% of total variance was attributable to stable inter-individual differences after accounting for fixed effects, supporting the appropriateness of mixed-effects modelling and the stability of DPOAE measurements across repeated assessments.

## 4. Discussion

### 4.1. Effect of Dental Procedures on Cochlear Function

The present study demonstrated significant reductions in DPOAE amplitudes immediately following routine dental procedures, with recovery observed at the 48-h follow-up. These findings indicate transient changes in cochlear outer hair cell (OHC) function consistent with acute noise exposer from dental equipment. This pattern aligns with previous research documenting short-term reductions in oto-acoustic emission amplitudes following occupational and recreational noise exposure [16,17,26,33]. The absence of a significant Frequency × Time interaction indicates that the observed amplitude reduction occurred broadly across the tested frequency range, a diffuse response pattern also noted in young adults with normal hearing following moderate noise exposure [17,26].

The noise generated by high-speed rotary instruments and ultrasonic scalers is broadband and intermittently intense [3,4]. Even brief exposure to such sound levels increases the metabolic demand on OHCs due to sustained electromotive activity required for cochlear amplification [17,18]. This elevated metabolic load can temporarily reduce the efficiency of the cochlear amplifier [20,21]. The subsequent recovery of DPOAE amplitudes within 48 h suggests that, for exposures within physiological tolerance, OHC function can be restored [21].

### 4.2. Personal Listening Device Use and Cochlear Susceptibility

Although the two groups differed in their reported recreational listening habits, baseline DPOAE amplitudes were comparable between those exposed only to occupational noise and those who also used personal listening devices (PLDs). This finding is consistent with evidence that young adults with habitual PLD use often present with clinically normal audiometric thresholds and preserved OHC function at rest [18,26,34]. However, a preserved baseline does not guarantee equivalent cochlear resilience under conditions of additional acoustic stress. Experimental studies suggest that repeated subclinical noise exposure can alter cochlear physiology without producing measurable baseline deficits [26,35].

In the present study, the significant Group × Time interaction supports this interpretation. Dental professionals who reported PLD use exhibited a different temporal pattern of DPOAE change following occupational noise exposure compared to those exposed to dental noise alone. This differential response—characterized by an altered trajectory of amplitude reduction or recovery—suggests that cumulative acoustic history may modulate the cochlea’s acute response to subsequent stress. This interpretation aligns with translational research demonstrating that prior acoustic exposure can modify cochlear response dynamics even when standard clinical measures remain normal [35].

### 4.3. Combined Occupational and Recreational Exposure Effects

The significant Group × Time interaction indicates that the temporal trajectory of DPOAE change following dental procedures differed according to the participants’ overall exposure profile. Crucially, because baseline DPOAE amplitudes were equivalent, this interaction cannot be attributed to pre-existing cochlear dysfunction in the PLD group. Rather, it suggests that a history of recreational noise exposure may have influenced the susceptibility or recovery behaviour of OHCs when challenged by occupational noise.

This finding contributes beyond confirming the transient effects of dental noise alone; it highlights that acoustic exposure history may be a relevant modifier of short-term cochlear responses. While recovery was observed within 48 h in this young cohort, such differential response dynamics raise important considerations for cumulative occupational risk over extended clinical careers. The results underscore the principle that ototoxic risk is not solely determined by the magnitude of a single exposure event, but also by the individual’s broader acoustic environment.

### 4.4. Healthcare and Occupational Implications

From an occupational medicine perspective, these findings demonstrate that routine dental procedures can induce measurable, albeit transient, alterations in cochlear function. Although reversible at the individual episode level, recurrent exposure throughout a clinical career may contribute to long-term auditory vulnerability. This is particularly relevant for professionals who also engage in frequent recreational listening, as their cochleae may experience a greater cumulative acoustic load.

The strong test–retest reliability of DPOAE measurements in this study supports their suitability for longitudinal occupational monitoring. As a direct indicator of cochlear amplifier integrity, DPOAEs can detect early, functional changes before they manifest as permanent threshold shifts on conventional audiometry. Integrating objective cochlear assessments into hearing conservation programs for dental professionals could therefore facilitate earlier identification of at-risk individuals and enable more timely preventive interventions.

Preventive strategies should be multi-faceted, including the optimisation of engineering controls (e.g., equipment maintenance to minimise noise output), administrative controls (e.g., structured noise-awareness education), and personal protective equipment where appropriate. Crucially, counselling on safe recreational listening practices should be integrated into occupational health guidance, addressing the cumulative nature of acoustic exposure across both professional and personal domains. These measures align with global initiatives aimed at reducing noise-related auditory risk within healthcare environments [1,2,22].

### 4.5. Limitations

Several limitations of this study warrant consideration. First, cumulative lifetime noise exposure was not quantified using personal dosimetry, which limits the precision of exposure-response modelling. Second, DPOAEs primarily reflect OHC function and do not directly assess synaptic or neural integrity structures now recognised as vulnerable to noise injury even in the absence of hair cell loss [35]. Third, the follow-up interval was limited to 48 h, preventing evaluation of longer-term adaptation or potential delayed effects. Finally, PLD use was self-reported, introducing the possibility of recall bias. Future longitudinal studies incorporating objective exposure measurement, extended follow-up periods, and broader audiological assessments (including electrophysiological measures of neural function) are needed to more fully characterise cumulative auditory risk in this population.

## 5. Conclusions

Routine dental procedures involving high-speed and ultrasonic instruments can induce transient reductions in distortion product otoacoustic emission amplitudes in young professionals with normal audiometric thresholds. These acute changes, evident immediately following exposure and recovering within 48 h, suggest a reversible metabolic effect on outer hair cells rather than immediate permanent damage. Importantly, the temporal pattern of this cochlear response appears to be modulated by an individual’s history of recreational noise exposure, highlighting the cumulative nature of acoustic stress.

Distortion product otoacoustic emissions provide a sensitive, objective, and reliable tool for detecting early cochlear functional changes that may precede abnormalities on conventional audiometry. Incorporating such objective monitoring into occupational hearing health surveillance could enhance the early identification of exposure-related cochlear stress and support the development of targeted preventive strategies for dental healthcare personnel.

## Data Availability

The data presented in this study are available on request from the corresponding author. The data are not publicly available due to privacy and ethical restrictions.
